# The Relationship of Age-Related Hearing Loss with Cognitive Decline and Dementia in a Sinitic Language-Speaking Adult Population: A Systematic Review and Meta-Analysis

**DOI:** 10.1093/geroni/igac078

**Published:** 2022-12-20

**Authors:** Xinxing Fu, Robert H Eikelboom, Rong Tian, Bo Liu, Shuo Wang, Dona M P Jayakody

**Affiliations:** Beijing Institute of Otolaryngology, Beijing Tongren Hospital, Capital Medical University, Beijing, China; Centre for Ear Sciences, Medical School, The University of Western Australia, Crawley, Western Australia, Australia; Department of Speech Language Pathology and Audiology, University of Pretoria, Pretoria, South Africa; Brain and Hearing, Ear Science Institute Australia, Subiaco, Western Australia, Australia; Centre for Ear Sciences, Medical School, The University of Western Australia, Crawley, Western Australia, Australia; Beijing Institute of Otolaryngology, Beijing Tongren Hospital, Capital Medical University, Beijing, China; Beijing Institute of Otolaryngology, Beijing Tongren Hospital, Capital Medical University, Beijing, China; Centre for Ear Sciences, Medical School, The University of Western Australia, Crawley, Western Australia, Australia; Brain and Hearing, Ear Science Institute Australia, Subiaco, Western Australia, Australia

**Keywords:** Age-related hearing loss, Cognitive decline, Dementia, Sinitic tonal language

## Abstract

**Background and Objectives:**

Substantial evidence supports the association between untreated hearing loss, cognitive decline, and dementia in the non-tonal language-speaking population. Whether a similar association between hearing loss and cognitive decline and dementia exists in Sinitic tonal language-speaking people is yet to be elucidated. We aimed to systematically review the current evidence on the association between hearing loss and cognitive impairment/decline, and dementia in older adults who speak a Sinitic tonal language.

**Research Design and Methods:**

This systematic review considered peer-reviewed articles that employed objective or subjective hearing measurement and cognitive function, cognitive impairment, or diagnosis of dementia. All articles written in English and Chinese and published before March 2022 were included. Databases including Embase, MEDLINE, Web of Science, PsycINFO and Google Scholar, SinoMed, and CBM were utilized using MeSH terms and keywords.

**Results:**

Thirty-five articles met our inclusion criteria. Of these, 29 unique studies with an estimated 372,154 participants were included in the meta-analyses. Among all included studies, the effect size of cognitive function with hearing loss, the regression coefficient was −0.26 (95% confidence interval [CI], −0.45 to −0.07). Among cross-sectional and cohort studies, a significant association was found between hearing loss and cognitive impairment and dementia, with odds ratios of 1.85 (95% CI, 1.59–2.17) and 1.89 (95% CI, 1.50–2.38), respectively.

**Discussion and Implications:**

Most of the studies included in this systematic review observed a significant association between hearing loss and cognitive impairment and dementia. There was no significant difference to the findings in non-tonal language populations.

**Translational Significance:** Hearing loss may be a modifiable solution for health problems associated with cognitive impairment. Steps should be taken to incorporate hearing assessment and cognitive screening in clinical protocols for older adults 60 years and older in both hearing and memory clinics.

Hearing loss affects more than 1.5 billion people worldwide, including 1.16 billion with mild hearing loss and 430 million people with moderate or higher levels of hearing loss, over 58% of which is experienced by adults above the age of 60 years (World Health Organization, 2021). Unaddressed hearing loss not only affects listening and communication (Vas, 2017), but also cognitive functioning (Strutt et al., 2022), and can result in social isolation and loneliness (Shukla et al., 2020) and mental ill-health (Jayakody et al., 2018a; Rutherford et al., 2018). Age-related hearing loss (ARHL) is linked to cognitive impairment or decline, according to evidence from cross-sectional (Deal et al., 2015; Gussekloo et al., 2005; Jayakody et al., 2018b) and longitudinal (Heywood et al., 2017; Lin et al., 2011) investigations.

Dementia is a condition in which a person’s cognitive functioning, including thinking, remembering, and reasoning, has deteriorated to the point where it interferes with daily living and activities. A total of 57.4 million people were affected by dementia worldwide in 2019, and this will increase to 152.8 million cases in 2050 (GBD Dementia Forecasting Collaborators, 2022). However, no disease-modifying treatments are currently available for adults with dementia; thus, an emphasis on risk factor reduction, particularly modifiable risk factors, is warranted. Midlife hearing loss is linked to an increased risk of dementia, contributing 8% of the modifiable risk factors (Livingston et al., 2020; Mukadam et al., 2019).

Most studies that investigated this association have been conducted on non-tonal language speakers. A meta-analysis that included nine cohort studies enrolled from five countries, including Australia, the United States, the United Kingdom, Germany, and the Netherlands, reported a significant association with hearing loss and cognitive impairment (odds ratio [OR] = 1.22) and dementia (OR = 1.28; Loughrey et al., 2018).

However, there are several differences between non-tonal and the Sinitic family of tonal languages, which means that generalizing the findings regarding the relationship between hearing loss and cognitive impairment from non-tonal language speakers to tonal language speakers should be done with caution. Approximately 50% of the world’s population speaks a tonal language, most of whom speak one of the Sinitic (Chinese) languages or dialects. Mandarin is the dominant dialect of Chinese and is considered the standard language in mainland China.

First, in Sinitic languages, the lexical meaning is conveyed by pitch (tone) variations at the monosyllabic level. In a non-tonal language, on the other hand, the meanings of words do not change when the pitch changes. The perception of tonal language can be compared to music perception, as evidenced by the fact that speaking tonal language improves pitch perception in music and vice versa (Ngo et al., 2016). Playing a musical instrument has been shown to improve various cognitive functions in the brain, including memory and executive functioning (Mansky et al., 2020). According to available psychophysiological evidence, a Sinitic tonal language background may also be related to increased general cognitive function, demonstrating that Cantonese speakers showed better working memory associated with pitch perception than English speakers (Bidelman et al., 2013).

Second, the speech spectrum of Sinitic languages differs from that of non-tonal languages (Hu et al., 2019). There is a significant disparity between Chinese and English on the band information function (BIF), which quantifies contributions among frequency regions (Chen et al., 2016). For example, most speech information in Mandarin is clustered between 0.5 and 2 kHz (Nicholas et al., 2021). Mandarin speakers may be less susceptible to age-related high-frequency hearing loss (Hu et al., 2019). Considering that the high-frequency hearing thresholds exhibit a decline at least a decade earlier than the midfrequency hearing thresholds (Salvi et al., 2018), compared to non-tonal language speakers, high-frequency ARHL may have less impact on the speech perception of tonal language speakers. However, it should be noted that factors such as education, occupation, and physical, social, and leisure activities also influence the cognitive reserve (Harrison et al., 2015).

In recent years, there has been an increase in the number of reports on the links between ARHL and cognitive impairment or dementia in Sinitic language speakers (Diao et al., 2021; Fu et al., 2021; Gao et al., 2020; Ma et al., 2021; Ren et al., 2019; Sun et al., 2021; Wang et al., 2022; Xu et al., 2021; Zhao et al., 2021). The majority of research has shown a link between hearing loss and cognitive impairment or dementia. One cross-sectional study reported that pure tone average (PTA) was negatively correlated with the Montreal Cognitive Assessment (MoCA) score in a Han Chinese population (Ren et al., 2019). Another longitudinal study reported that the risk of incident cognitive impairment over a 6-year follow-up was 1.9-fold higher for participants developing hearing loss than those without (Chen & Lu, 2020). However, it is not clear whether the relationship between hearing loss and cognitive impairment/decline or dementia in the Sinitic language-speaking population reflects the results found in non-tonal language speakers (Ford et al., 2018; Loughrey et al., 2018). A systematic review and meta-analysis for studies on Sinitic language-speaking populations may clarify this; to the best of our knowledge, this has not yet been conducted and reported.

The objective of this research was to systematically review the current evidence on the association between ARHL and cognitive function, cognitive impairment, and/or dementia in adult Sinitic language speakers and conduct a meta-analysis of the published evidence.

## Method

This current systematic review conformed to the Preferred Reporting Items for Systematic Review and Meta-Analysis (PRISMA) statement and was registered in the International Prospective Register of Systematic Reviews (PROSPERO; Registration number: CRD42021235310).

### Study Eligibility

We included randomized controlled trials, cohort studies, cross-sectional, and case-control studies investigating the association between hearing loss and cognitive impairment/decline or dementia in Sinitic language-speaking populations. Studies were included if: (a) the participants of the study were aged 40 years and older; (b) hearing loss was self-reported or measured by pure tone audiometry and/or speech test; (c) they documented cognitive impairment, including global cognition assessment or verbal and nonverbal measurements of specific cognitive domains, or a clinical diagnosis of dementia. Studies were excluded if: (a) they included participants with a previous cognitive/neurological disorder, for example, preexisting intellectual disability, and acquired brain injury; or (b) a control group, for example, participants without cognitive impairment, was not included in the study.

Primary measures of cognitive function or cognitive impairment, or diagnosis of dementia were (a) global cognition assessment tools, including MoCA or MMSE (Mini-Mental State Examination), (b) both verbal and nonverbal measurements of specific cognitive domains, including attention, immediate or delayed recall, speech fluency, processing speed, reasoning, visuospatial ability, working or semantic memory, or (c) a clinical diagnosis of dementia. Primary measures of hearing were pure tone audiometry, speech reception thresholds, or subjective hearing loss assessment, including self-report.

### Information Sources

The search was carried out in the following databases: Embase, MEDLINE, Web of Science, and PsycINFO. In addition, SinoMed and the Chinese Biomedical Database (CBM) were used to obtain the Chinese language reports. A further gray literature search was conducted using Google Scholar to identify relevant articles not found through the database search. References and citations of relevant publications identified for inclusion and reviews on this topic were scrutinized. English and Chinese language publications were included.

### Search Strategy

Both Medical Subject Headings (MeSH) terms and keywords were utilized to retrieve as many relevant articles as possible in EMBASE and MEDLINE. Keywords and their synonyms, abbreviations, and truncations were used in the Web of Science, PsycINFO, and Google Scholar. A similar strategy was used for the two Chinese databases, SinoMed and CBM. The search terms were divided into three domains: (a) hearing loss, (b) cognitive decline, and (c) Sinitic tonal language. Two independent reviewers were deployed to undertake the search and the processes of identification of studies to minimize personal errors and system biases. The detailed search strategy was described in the published protocol (Fu et al., 2022).

### Data Management and Study Selection

All results from database and gray searches were imported into Rayyan (www.rayyan.ai), an online organizational tool for systematic reviews. Study selection: The selection of the articles was carried out in two phases; first, the titles and abstracts were screened by the independent reviewers based on the eligibility criteria for further review; second, the full texts of the eligible articles were analyzed based on the eligibility criteria. The search and screening of the publication were conducted independently by two researchers (X. Fu and R. Tian) for both English and Chinese articles. The discussion resolved any disagreement until consensus was reached or in consultation with other authors (D. M. P. Jayakodi, R. E. Eikelboom, and S. Wang).

### Data Extraction

The information extracted from the included articles included: (a) authors and year of publications; (b) location of the study (countries and cities); (c) demographics of the participants, for example, age, sex, language; (d) method of primary measures in hearing or cognitive function; (e) outcomes of primary measures; and (f) significant findings (main results). Regression coefficients or odds ratios between ARHL and cognitive decline or dementia were recorded.

### Quality Assessment and Meta-Bias(es)

The quality of evidence reported in the included studies was assessed using the Newcastle–Ottawa Scale to determine the risks of bias. Articles were evaluated based on eight criteria organized into three domains: selection, comparability, and outcome. The detailed protocol for quality assessment was described in the Supplementary Materials (Tian et al., 2021), see Supplementary Table 1. The review excluded any articles that were evaluated as having poor quality. Publication bias was assessed through a funnel plot. The sensitivity analysis evaluated selective reporting.

### Meta-Analytic Approach

For continuous variables, the regression coefficient was chosen as a measure of the effect size of the linear association between hearing loss and cognitive function. Negative scores indicated that greater hearing loss was associated with poorer cognition. For binary dependent variables, odds ratio, relative ratio, or hazard ratio were used by the included studies to assess the association between hearing loss and cognitive impairment or dementia. This review uses the generic term “odds ratio” to describe the odds ratio, relative risk, or hazard ratio reported by the individual studies. Results that were adjusted for confounders were used when available. Separate analyses were undertaken for different study designs (including cross-sectional, case–control, and cohort). A random-effects model was utilized due to the potential study heterogeneity. The *I*^2^ statistic was used to assess the heterogeneity of the studies. A *p*-value < .05 was considered to indicate a significant effect. Stata 17.0 was used for the meta-analyses (StataCorp LLC, 2021) using the *meta*-command.

### Synthesis of Results

The data extracted from the articles were tabulated to show the overall quality and the main findings. The results were analyzed based on hearing loss, cognitive decline, and impairment to synthesize the evidence.

### Heterogeneity and Sensitivity Analyses

*I* ^2^ tests were conducted to test heterogeneity. The results of *I*^2^ test were utilized to quantify heterogeneity. Heterogeneity with *I*^2^ values below 40% is considered as low, 41%–60% as medium, and over 60% as high. Heterogeneity was further investigated by subgroup and sensitivity analyses. Sensitivity analysis was conducted by repeating the meta-analysis when omitting individual studies.

## Results

### Study Selection

**Figure 1.**
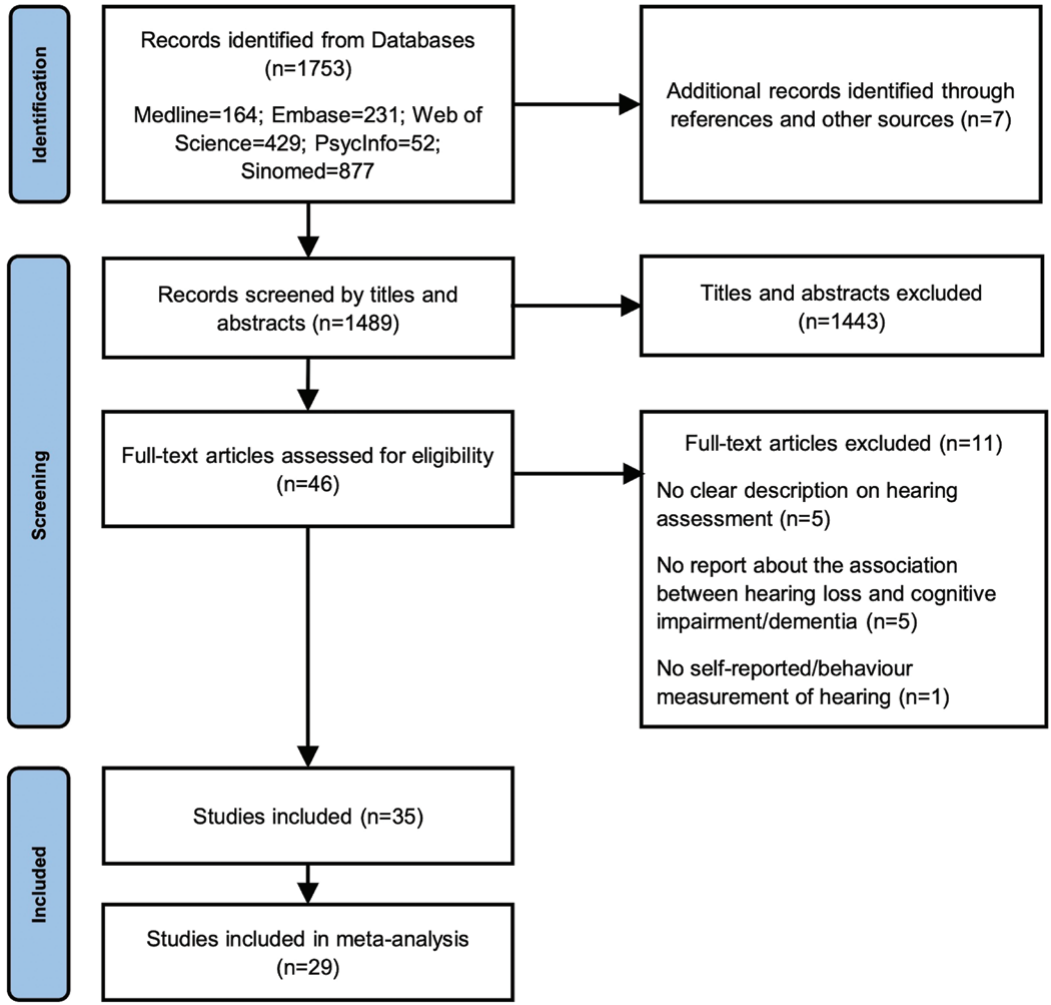
PRISMA flow chart showing the results of the systematic literature search. PRISMA = Preferred Reporting Items for Systematic Review and Meta-Analysis.

The results of the systematic literature search are summarized in Figure 1. One thousand seven hundred fifty-three articles were screened for eligibility, and 35 articles were found to meet the criteria for inclusion, including three case–control studies, 24 cross-sectional studies, and eight longitudinal studies. Eight of these included articles were published in Chinese, and the others in English. Six of these studies lacked adequate data for a meta-analysis (Diao et al., 2021; Ma et al., 2021; Nicholas et al., 2021; Qiu et al., 2020; Ren et al., 2020; Wang et al., 2019). The quantitative analysis included 29 studies with three case–control studies, 19 cross-sectional, and seven longitudinal studies, containing 2,854, 316,132, and 53,168 participants, respectively.

### Study Characteristics

Table 1 summarizes the 35 included studies, among which three studies were divided into two substudies each, by gender or cognitive impairment/dementia (Pan et al., 2021a; Wang et al., 2020a, 2021a). Fourteen studies used validated audiology methods to measure hearing, including pure-tone audiometry and speech reception thresholds. Twenty studies identified hearing loss through simple self-reported questions (e.g., Do you have any difficulty with your hearing?). One study utilized International Classification of Diseases (ICD-9) codes to determine the hearing loss (Su et al., 2017). The definition of hearing loss from the World Health Organization was generally used by most studies using audiometry thresholds; however, the octave frequencies tested ranged from 500 to 4000 Hz, 120 to 8000 Hz, and 250 to 8000 Hz, while several studies used the hearing thresholds at 3000 Hz and 6000 Hz as well.

**Table 1.** Summary of Included Studies

Study name	Study design	Participants	Hearing assessment		Cognitive assessments		The measure of risk in meta-analysis
			Method	Criteria	Method	Criteria	
Chen (2021b)	Longitudinal	Four thousand two hundred sixty-seven community-dwelling people aged 65≤ in China	Self-reported simple question	Question: “Do you have any difficulty with your hearing?”	CMMSE	Scored <18 was considered cognitively impaired	OR = 1.59 (1.26, 1.92)
Chen and Lu (2020)	Longitudinal	Six thousand three hundred nine community-dwelling people aged 65≤ in China	Self-reported simple question	Question: “Do you have any difficulty with your hearing?”	CMMSE	Scored <18 was considered cognitively impaired	RR = 1.90 (1.69, 2.14)
Chen and Zhou (2020)	Longitudinal	Nine thousand six hundred seventy-nine community-dwelling people aged 65≤ in China	Self-reported simple question	Question: “Do you have any difficulty with your hearing?”	CMMSE	Scored <18 was considered cognitively impaired	OR = 2.93 (2.61, 3.30)
Chen and Zhou (2022)	Cross-sectional	Eleven thousand seven hundred twenty-two community-dwelling people aged 65≤ in China	Self-reported simple question	Question: “Do you have any difficulty with your hearing?”	CMMSE	Scored <18 was considered cognitively impaired	OR = 2.05 (1.75, 2.40)
Chen (2021a)	Longitudinal	Ten thousand three hundred forty-one community-dwelling people aged 65≤ in China	Subjective validated by examiner	If participants were not able to hear the questions at all or partly	CMMSE	Scored <18 was considered cognitively impaired	HR = 1.42(1.28, 1.58)
Dai et al. (2021)	Case–control	Two hundred outpatients with hearing loss aged 60≤ in Shandong	Pure tone audiometry	Continuous variable 0.5, 1, 2, and 4 kHz/better ear	MoCA	MoCA < 26	OR = 1.14 (0.85, 2.22)
Diao et al. (2021)	Cross-sectional	Two hundred one community-dwelling people aged between 60 and 69 in Beijing	Pure tone audiometry	0.5, 1, 2, 3, 4, 6, and 8 kHz	MoCA	N/A	Not included in meta-analysis
Fu et al. (2021)	Cross-sectional	Two hundred ninety-three community-dwelling people aged 60≤ in Beijing	Pure tone audiometry	N/A 0.5, 1, 2, 3, 4, 6, and 8 kHz	CANTAB (PAL, DMS, SWM), MoCA-HI	N/A	Regression β = −0.04 (−0.05, −0.02)
Gao et al. (2020)	Longitudinal	Eight thousand eight hundred forty-four community-dwelling people aged 65≤ in China	Self-reported simple question	Question: “Do you have any difficulty with your hearing?”	CMMSE	Scored <18 was considered cognitively impaired	OR = 2.48 (1.22, 5.06)
Guo et al. (2020)	Cross-sectional	Three thousand nine hundred ninety-six community-dwelling people aged 60≤ in Sichuan	Self-reported	Not described	AD8; CMMSE	Considered cognitively impaired if MMSE ≤ 17 for illiterates; ≤20 for primary school graduates; and ≤24 for junior high school or above	OR = 1.70 (1.06, 2.75)
Han et al. (2019)	Cross-sectional	Two thousand seventeen community-dwelling people aged 60≤ in Beijing	Self-reported	Not described	CMMSE	The thresholds for those who were illiterate, or attended at most primary school, middle school, or universities were ≤17, 17–20, 21–22, and 23–24, respectively	OR = 1.68 (1.23, 2.30)
Hung et al. (2015)	Case–control	Two thousand four hundred forty (case group: 488 with diagnosed AD; control group: 1,952 without AD)	Pure tone audiometry	ICD-9 codes 388.01, 389.0, 389.1, 389.2, 389.8, and 389.9	ICD-9 AD	Dementia	OR = 1.39 (1.05, 1.84)
Li et al. (2017)	Cross-sectional	Three hundred eighty-five community-dwelling people with chronic diseases aged 80≤ in Anhui	Self-reported	Not described	CMMSE	Scored less than 26 points were considered as cognitive impairment	OR = 2.01 (1.17, 3.47)
Luo et al. (2018)	Cross-sectional	Two hundred fifty thousand seven hundred fifty-two community-dwelling people aged 65≤ in China	Pure tone audiometry	>40 dB in the better ear 0.5, 1, 2, 4 kHz	ICD-10	Dementia	OR = 1.04 (0.88, 1.22)
Ma et al. (2017)	Cross-sectional	Five thousand seven hundred eight community-dwelling people aged 60≤ in China	Self-reported	Not described	MMSE and physical frailty	Cutoffs for those who were illiterate, or attended at most primary school, middle school, or university were ≤17, ≤20, ≤22, and ≤24 respectively	Not included in the meta-analysis
Ma et al. (2020)	Longitudinal	One thousand one hundred seventeen community-dwelling people aged 70≤ in Jiangsu	Self-reported	Not described	CMMSE	Considered cognitively impaired if MMSE ≤ 17 for illiterates; ≤20 for primary school graduates; and ≤24 for junior high school or above	OR = 1.51 (1.06, 2.15)
Mukadam et al. (2019)	Cross-sectional	Two thousand one hundred sixty-two community-dwelling people aged 65≤ in China	Self-reported	Not described	Not described	Dementia	RR = 1.90 (1.40, 2.70)
Nicholas et al. (2021)	Cross-sectional	Two hundred thirty-eight community-dwelling people aged 55≤ in Singapore	Pure tone audiometry		CANTAB (PAL, VRM, MTT, DMS, SWM)	NA	Not included in the meta-analysis
Pan et al. (2021a)	Cross-sectional	Eight hundred forty-nine community-dwelling people aged 60≤ in Shanghai	Self-reported	Not described	MoCA-Basic	Considered cognitively impaired if MoCA-B ≤ 19 for education years ≤6; ≤22 for education years 7–12; and ≤24 for education years >12	OR = 2.38 (1.67, 3.30)
Pan et al. (2021b)	Cross-sectional	Seven hundred thirty-four community-dwelling people aged 60≤ in Shanghai	Self-reported	Not described	MoCA-Basic	Not described	AD: OR = 3.13 (1.46, 6.66) MCI: OR = 2.25 (1.55, 3.26)
Qiu et al. (2020)	Longitudinal	Three thousand eight hundred fifty-nine community-dwelling people aged 65≤ in China	Self-reported simple question	Question: “Do you have any difficulty with your hearing?”	CMMSE	Not described	Not included in the meta-analysis
Ren et al (2019)	Cross-sectional	Eighty community-dwelling people aged 60≤ in Shandong	Pure tone audiometry	0.125, 0.25, 0.5, 1, 2, 4, and 8 kHz/both ears, Speech reception thresholds	AVLT, Stroop, SDMT TMT-A, TMT-B, MoCA	N/A	Regression β = −0.05 (−0.08, −0.02)
Ren et al. (2020)	Cross-sectional	One hundred thirty-one outpatients with hearing loss aged 60≤ in Shanghai	Pure tone audiometry	0.5, 1, 2, and 4 kHz; speech reception rate	CMMSE	Continuous variable	Not included in meta-analysis
Rong et al. (2020)	Cross-sectional	Eighteen thousand thirty-eight community-dwelling people aged 45≤ in China	Self-reported simple question	Question: “Is your hearing excellent, very good, good, fair, or poor?”	Word recall test, TICS, Pentagon drawing test	N/A	Regression β = −0.43 (−0.55, −0.31)
Su et al. (2017)	Longitudinal	Eight thousand one hundred twenty-one community-dwelling people in Taiwan	Pure tone audiometry	ICD-9-CM codes 389.10–389.12 and 388.01	Dementia	ICD-9	HR = 1.30 (CI = 1.14, 1.49)
Sun et al. (2021)	Cross-sectional	Three thousand seven hundred twenty-one community-dwelling people aged 65≤ in China	Self-reported simple question	Question: “Do you have any difficulty with your hearing?”	CMMSE	N/A	Regression β = −0.73, *SE* = 0.12
Wang et al. (2019)	Cross-sectional	Thirty-one outpatients with hearing loss aged between 52 and 75 in Beijing	Pure tone audiometry	Four frequencies average >25 dB HL 0.5, 1, 2, and 4 kHz/better ear	CANTAB (PAL, DMS, SWM)	N/A	Not included in the meta-analysis
Wang et al. (2020b)	Cross-sectional	One thousand two hundred fifty community-dwelling people aged 65≤ in villages of China	Self-reported	Good, moderate, poor	CMMSE	Considered cognitively impaired if scored ≤17 for illiteracy, ≤20 for primary school, and ≤24 for secondary school	Male: OR = 2.02 (1.21, 3.39) Female: OR = 2.11 (1.32, 3.37)
Wang et al. (2020a)	Cross-sectional	One thousand two hundred fifty community-dwelling people aged 65≤ in villages of China	Self-reported	Good, moderate, poor	CMMSE	Considered cognitively impaired if scored ≤17 for illiteracy, ≤20 for primary school, and ≤24 for secondary school	OR = 0.39 (0.30, 0.51) poor hearing as reference
Wang et al. (2021b)	Cross-sectional	Three thousand seventy-five community-dwelling people aged 60≤ in Hebei	Pure tone audiometry	Either ear for a high-frequency hearing loss >25 dB	MoCA, MMSE	MCI diagnosis criteria	OR = 1.49 (1.14, 1.93)
Wang et al. (2021a)	Cross-sectional	One thousand twelve community-dwelling people aged 60≤ in Shanghai	Pure tone audiometry	>40 dB loss in the better ear 0.25, 0.5, 1, 2, 4, and 8 kHz Speech frequency; high frequency	CMMSE	CI was defined as MMSE ≤ 17 for illiterates; for primary school graduates; and ≤24 for junior high school graduates or above	Female: OR = 2.92 (1.67, 5.12) Male: OR = 2.56 (1.25. 5.23)
Wang et al. (2022)	Cross-sectional	One hundred forty-five community-dwelling people aged 60≤ in Shandong	Pure tone audiometry	PTA (range: 0.5–4 kHz) of the better ear ≥20 dB hearing loss	Stroop, AVLT, SDMT, TMT, MoCA	N/A	Regression β = –0.31 (–0.14, −0.05)
Xu et al. (2021)	Cross-sectional	Seven hundred thirty-seven community-dwelling people aged 60≤ in Tianjin	Pure tone audiometry	N/A 0.5, 1, 2, 3, 4, 6, and 8 kHz	CMMSE	N/A	Regression β = −0.52, *SE* = 0.20
Yu and Woo (2019)	Longitudinal	Two thousand two hundred fifty-nine community-dwelling people aged 60≤ in Hong Kong	Self-reported	Question: “Do you have any difficulty with your hearing?”; very good and good = robust; fair, not too well, poor, and very poor = poor	Five-item AMIC	AMIC score ≥ 3 is predictive of MCI	OR = 2.2 (1.8, 2.8)
Yu et al. (2020)	Case–control	Two hundred fourteen outpatients with hearing loss aged 60≤ in Jiangsu	Pure tone audiometry	NA 0.5, 1, and 2 kHz/better ear	MoCA	N/A	Regression β = −2.26, *SE* = 0.30
Zhao et al. (2021)	Cross-sectional	Thirteen thousand nine hundred fourteen community-dwelling people aged 45≤ in China	Self-reported simple question	Question: “Do you have hearing problems?”	TICS battery	N/A	Regression β = −0.04 (−0.08, −0.04)

*Notes*: AD = Alzheimer’s disease; AMIC = Abbreviated Memory Inventory for the Chinese; AVLT = Auditory Verbal Learning Test; CANTAB = Cambridge Neuropsychological Test Automated Battery; CMMSE = Chinese version of the Mini-Mental State Examination; DMS = delayed matching to sample; HL = hearing level; HR = hazard ratio; ICD = International Classification of Diseases; MCI = mild cognitive impairment; MoCA = Montreal Cognitive Assessment; MTT = multitasking task; N/A = not applicable; OR = odds ratio; PAL = paired associates learning; PTA = pure tone average; RR = relative risk; SDMT = Symbol Digit Modalities Test; *SE* = standard error; SWM = spatial working memory; TICS = Telephone Interview of Cognitive Status; TMT = trail-making test; VRM = verbal recognition memory.

Cognitive function, cognitive impairment, or clinically diagnosed dementia were assessed in the included studies. For cognitive function assessment, except for global cognition, for example, MMSE or MoCA scores, participants’ specific domains of cognition were assessed by a series of tests in two studies (Ren et al., 2019; Wang et al., 2022), including verbal learning and memory, attention, psychomotor speed, and executive control were tested Stroop Color–Word Interference Test (Stroop), Auditory Verbal Learning Test, Symbol Digit Modalities Test, and Trail-Making Test. Besides these, a nonverbal-based cognition assessment using the Cambridge Neuropsychological Test Automated Battery (CANTAB) was reported in three studies (Fu et al., 2021; Nicholas et al., 2021; Wang et al., 2019).

For the evaluation of cognitive impairment, the assessment tool used by most researchers (in 21 studies) was the Chinese version of the Mini-Mental State Examination (C-MMSE). The cutoff criteria of C-MMSE varied among studies; a score below 18 on the MMSE was considered cognitive impairment in four studies (Chen, 2021b; Chen & Zhou, 2020, 2022; Gao et al., 2020). The cutoff score was adjusted for education level in nine studies, for example, 17 for illiteracy, 20 for primary school, and 24 for secondary school and above. Ten studies used the Chinese version of the Montreal Cognitive Assessment (C-MoCA). Two studies used the MoCA-basic version (40, 46), and one used the MoCA-hearing impairment version (Fu et al., 2021). Other cognitive assessments included Telephone Interview of Cognitive Status (TICS-10; Zhao et al., 2021), and the five-item Memory Inventory for the Chinese (Yu & Woo, 2019). Furthermore, four studies adopted the clinically diagnosed dementia or Alzheimer’s disease guidelines, ICD-9 or ICD-10 (Hung et al., 2015; Luo et al., 2018; Mukadam et al., 2019; Su et al., 2017).

### The Effect Size of Pooled Studies

**Figure 2.**
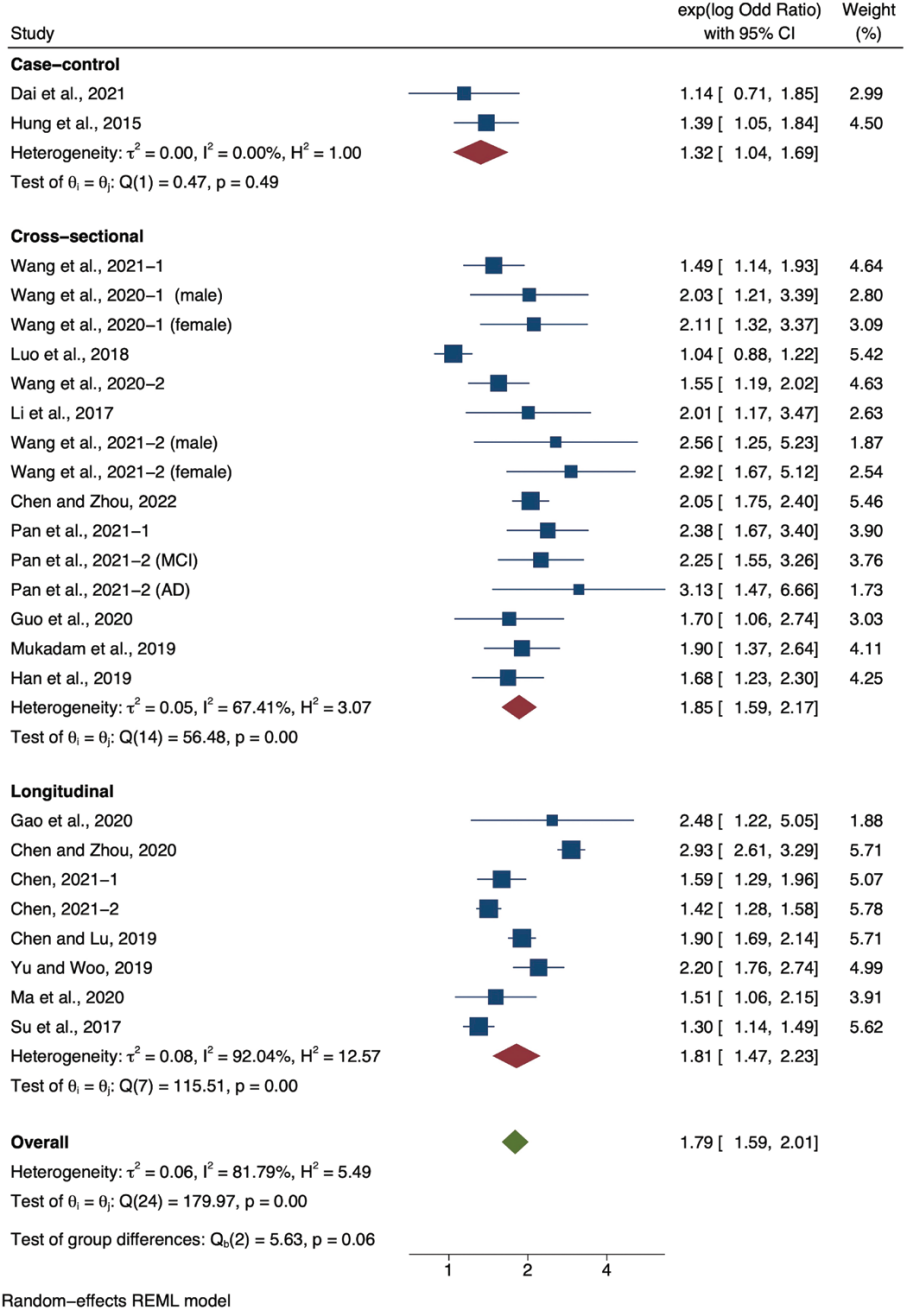
Forest plot showing the overall relationship between hearing loss and cognitive impairment with odds ratio as the effect size, grouped by case–control, cross-sectional studies, and longitudinal studies. AD = Alzheimer’s disease; CI = confidence interval; MCI = mild cognitive impairment.

Two types of effect sizes were used: the regression coefficient between hearing thresholds and cognition for continuous dependent variables, and the odds ratio between hearing loss and cognitive impairment or dementia for binary dependent variables. Among the 29 studies that were included in the meta-analysis, 18 reported odds ratios, one study (Su et al., 2017) reported hazard ratios, and two studies (Chen & Lu, 2020; Mukadam et al., 2019) reported relative risk ratios. The odds ratio of one study used in the meta-analysis was calculated using demographic data reported by the studies (Wang et al., 2020a). According to their study design, all other studies were adjusted for age, gender, and other factors. The pooled odds ratio of cognitive impairment/dementia of people with hearing loss across all studies was 1.82 (random-effects, 95% confidence interval [CI] = 1.61 to 2.05; Figure 2). Follow-up periods for the eight longitudinal studies varied, with three years of follow-up reported by six studies, 10 years by one study (Su et al., 2017), and one year by one study (Yu & Woo, 2019).

**Figure 3.**
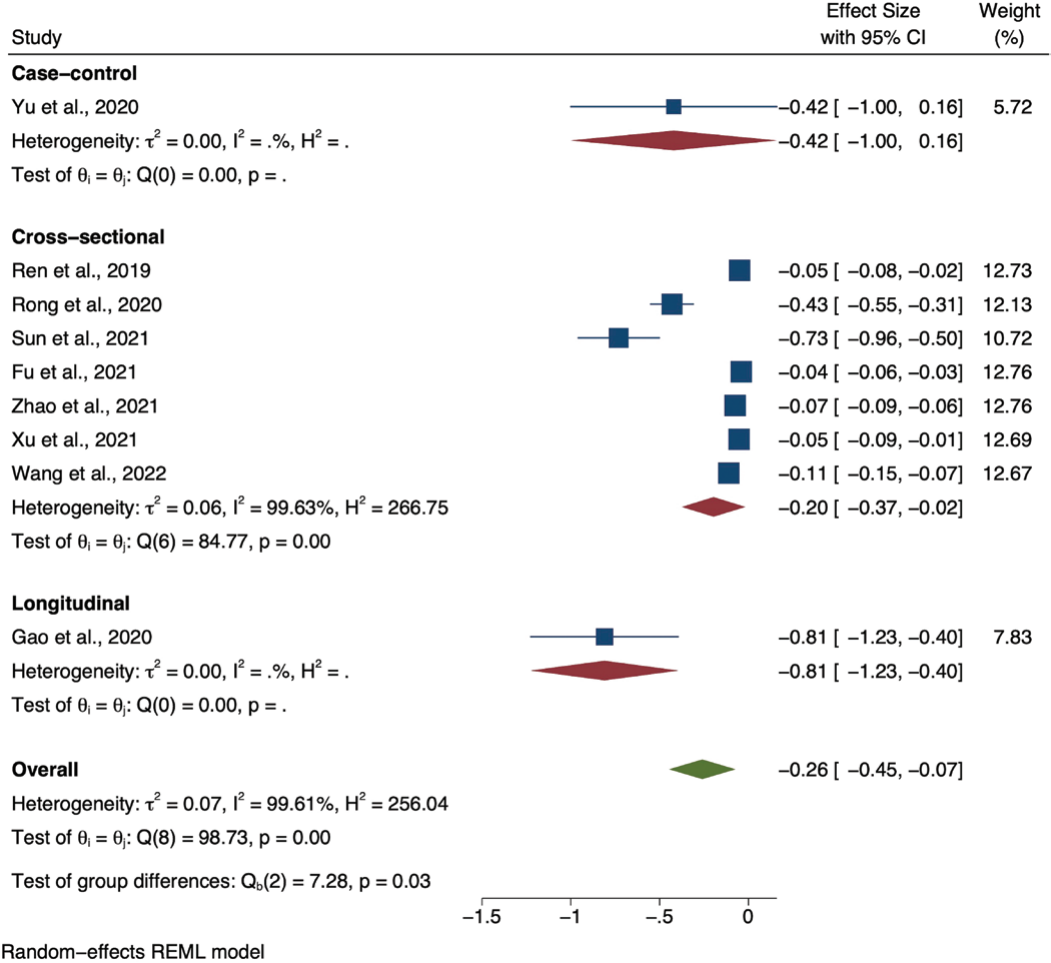
Forest plot showing the overall relationship between hearing loss and cognitive functions with the regression coefficient as the effect size, grouped by case–control, cross-sectional studies, and longitudinal studies. CI = confidence interval; REML = restricted maximum likelihood.

Nine studies assessed the cognitive function of participants, for which the regression coefficient and 95% CI were reported. Five of these studies reported hearing loss by audiometry, and four used self-reported questions. The pooled effect size (regression coefficient) between hearing loss and cognition across all studies was −0.26 (random-effects, 95% CI = −0.45 to −0.07), and −0.06 (95% CI = −0.09 to −0.03) in studies using audiometry, compared with −0.48 (95% CI = −0.81 to −0.15) in those with self-reported (Figure 3).

### Risk of Bias Within and Across Studies

**Figure 4.**
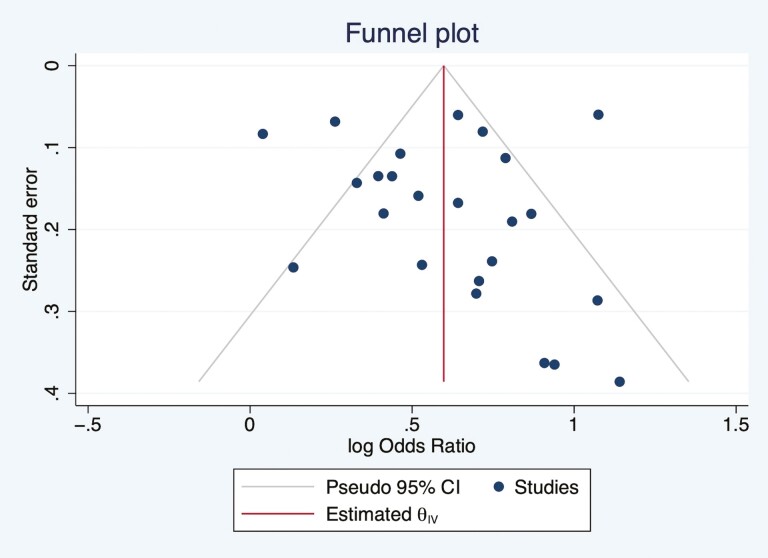
Funnel plot showing the distribution of studies with odds ratio as the effect size. CI = confidence interval.

**Figure 5.**
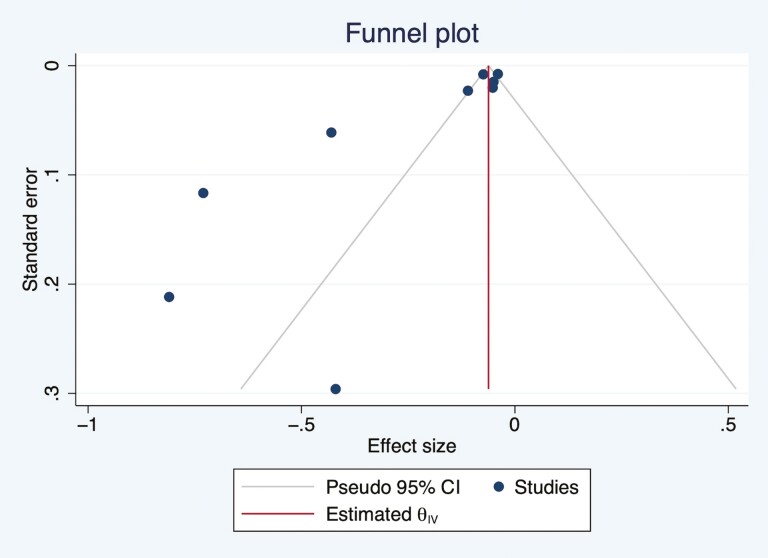
Funnel plot showing the distribution of studies with the regression coefficient as the effect size. CI = confidence interval.

According to the Newcastle–Ottawa Scale, the quality of all the studies included in the review was either good or fair (Supplementary Tables 1–3). High heterogeneities were present in the meta-analysis of cross-sectional and longitudinal studies with OR as the effect size, *I*^2^ = 67.41% and 90.73%, respectively. The heterogeneity was also high in pooled studies with regression coefficient as effect size, *I*^2^ = 99.61%. Publication bias was assessed through a funnel plot (Figures 4 and 5), which indicated that population bias and heterogeneity might be an issue in the meta-analysis, with positive studies more likely to be published, especially for the pooled analyses with OR as the effect size.

### Sensitivity Analyses

For all included studies, sensitivity analyses were conducted to examine the impact of a single study on the overall outcome of the meta-analysis. The meta-analysis was repeated by omitting individual studies for setting regression coefficient and OR as the effect size (Supplementary Figures 6 and 7). The removal of no single study resulted in a significant effect on the overall effect.

### Subgroup Analyses

Subgroup analyses were conducted for all the included studies based on the following: methods used to assess hearing (audiometry or reported-hearing loss) and cognition (cognitive impairment or diagnosed dementia), the sample size (<1,000, or 1,000–2,000, or >2,000), and the type of studies (cross-sectional or longitudinal; Supplementary Figures 1–5). The association between hearing loss and cognitive impairment/dementia still showed significance, although heterogeneity varied across the different sample size categories. The least heterogeneity (71.16%) was found in studies with less than 1,000 participants. The effect size of the association was more apparent in the studies with participants of less than 1,000 (odds ratio [OR] = 1.94). However, only a few studies with 1,000 to 2,000 participants (Supplementary Figure 3) were included in this review. Regarding the subgroup analysis by the types of hearing assessment, the effect size in studies using audiometry was much smaller, 1.47, compared to those using self-reported hearing loss, 1.96 (Supplementary Figure 2). Furthermore, the effect size of pooled studies on cognitive impairment was much larger than diagnosed dementia, 1.95 versus 1.45 (Supplementary Figure 1).

Similar results were found for the pooled studies with regression coefficient as the effect size. For the subgroup analysis by the types of hearing assessment, the effect size in studies using audiometry was much smaller, −0.06, compared to those using self-reported hearing loss, −0.48 (Supplementary Figure 4). The effect size of studies with participants of more than 2,000 was much higher than those of less than 1,000, −0.48 versus −0.06 (Supplementary Figure 5).

## Discussion

We provide a systematic review and meta-analysis of both cross-sectional and longitudinal, observational studies in Sinitic language-speaking populations looking at the relationship between hearing loss, cognitive function, and cognitive impairment/dementia. Except for two (Dai et al., 2021; Luo et al., 2018), most of the included studies observed a significant association between hearing loss and cognitive impairment or dementia. We found that the risk of cognitive impairment or dementia is greater among older people with hearing loss than those without, regardless of study design, method of assessment of hearing and cognition, and sample size. These findings are consistent with the findings of previous systematic reviews and meta-analyses of studies in non-tonal language populations (Ford et al., 2018; Lau et al., 2021; Taljaard et al., 2013). Furthermore, a similar association was found in eight included studies between hearing thresholds and cognitive function (impairment or not), except for in one study (Yu et al., 2020), in which a significant relationship was not observed. In other eight studies, participants with higher hearing thresholds tend to have the worse cognitive function (as a continuous variable), which also accords with an earlier published meta-analysis of non-tonal language participants (Loughrey et al., 2018).

It should be noted that the data analyzed in four of the eight longitudinal studies were derived from the same cohort, the Chinese Longitudinal Healthy Longevity Survey (CLHLS), which was the first and largest cohort study in a low and middle-income country (Yi, 2008). The CLHLS covered 22 provinces (out of 31 provinces) in China (Gu, 2007). The first-wave data were collected in 1998. Follow-up and recruitment of new participants were done in 2000, 2002, 2005, 2008, 2011, 2014, and 2018, respectively. However, the four studies analyzed the CLHLS data in different methods. For example, one study excluded 1,000 participants with cognitive impairment at baseline (Chen, 2021b). The odds ratio of cognitive impairment in people with self-reported hearing loss was 1.59, in the CLHLS study, excluding participants with cognitive impairment at baseline, lower than the odds ratios of 2.48 and 2.93 in the population without excluding baseline cognitive impairment.

One fundamental limitation among the included studies is the variability in methods by which the hearing loss was measured. First, assessments of hearing loss subjectively or imprecisely would probably reduce the accuracy of the association between hearing loss and cognitive impairment. Twenty studies relied on subjective reporting of hearing loss; among them, 16 studies reported the odd ratios of cognitive impairment or dementia, and four reported the regression coefficients between hearing sensitivity and cognitive function. This is a fast method in a community setting for identifying hard-of-hearing individuals, although studies have revealed that subjective hearing assessments have been valid (Bagai et al., 2006). The prevalence of hearing impairment tends to be underestimated by self-report in older adults aged 75 years and older, so false negatives of hearing impairment may affect the association between hearing loss and cognitive impairment. Only 10 studies adopted the pure tone audiometry to assess hearing impairment or sensitivity, which provides an accurate measurement of an individual’s hearing level, including five studies reporting the odds ratio and five studies reporting the regression coefficients; all of which reported smaller effect size compared to those using self-reported hearing.

Second, the cognitive assessment tools varied among the included studies, therefore, it is difficult to compare the outcomes directly. However, the MMSE, MoCA, and Abbreviated Memory Inventory for the Chinese (AMIC) were used in most of the studies, but it was not always stated whether they considered the hearing ability of the participants. By using verbal language instructions, it is possible that the hearing loss of the participants had an impact on their cognitive performance, causing an overestimate of the degree of cognition impairment (Dupuis et al., 2015). However, in one study, the MoCA for the hearing impaired was used, which was converted into a timed PowerPoint presentation, with all the verbal instructions replaced with visual instructions (Fu et al., 2021). Nonverbal cognition was assessed in three studies using the CANTAB (Fu et al., 2021; Nicholas et al., 2021; Wang et al., 2019).

In this meta-analysis, the effect size of pooled studies on dementia was 1.45 (95% CI, 1.11–1.91), which is in accord with a previously published finding of 1.38 (95% CI, 1.23–1.53) in the non-tonal language-speaking population (Ford et al., 2018). The effect size of pooled studies on cognitive impairment was 1.95 (95% CI, 1.73–2.18), which is consistent with a previously published meta-analysis of studies conducted in non-tonal language-speaking populations, which showed that the pooled odds ratio of cognitive impairment in people with hearing loss was 2.00 (95% CI, 1.39–2.89; Loughrey et al., 2018). However, in a more recent meta-analysis of studies conducted in non-tonal language-speaking populations, the OR of 1.44 (95% CI, 1.27–1.64; Lau et al., 2021) is significantly lower than reported in the present study. One possible explanation is that only studies of participants with mild cognitive impairment were included in this meta-analysis (in the non-tonal language population). On the other hand, in our analysis, there were no restrictions on the degree of cognitive impairment, and only one study utilized a two-stage diagnostic criteria for mild cognitive impairment (Wang et al., 2021b). All the others evaluated cognitive impairment by setting a cutoff value for the MMSE score, with some of them adjusting for education, as shown in Table 1.

While this analysis did confirm the association between hearing loss and cognitive decline and dementia in tonal language speakers, it did not find that speaking a tonal language was a protective for cognitive impairment as postulated in the study rationale. It is possible that the influence of speaking a tonal language on cognitive functioning is too small for studies of this nature to measure. If indeed a tonal language is protective of specific cognitive function, for example, working memory (Bidelman et al., 2013), then studies that include general measures of cognitive functioning, for example, CMMSE, and MoCA, are not sensitive enough to assess these. There was not sufficient data in the included studies for this to be examined; only four studies included a comprehensive test battery (Fu et al., 2021; Nicholas et al., 2021; Ren et al., 2019; Wang et al., 2022) that reported, for example, working memory. It is also important for studies to be aware of other potential factors that may affect the association between hearing loss and cognitive impairment, for instance, ethnicity (Golub et al., 2017), physical or leisure activities (Ma et al., 2021), and social isolation (Chen & Zhou, 2020). Participation in physical or leisure activities, living arrangements and family life, and attitudes to disability have strong cultural associations (Saravanabhavan & Saravanabhavan, 2001). Therefore, these confounding factors should be considered in future studies.

Most studies with a large sample size analyzed available data from existing population studies, like China Health and Retirement Longitudinal Study (Zhao et al., 2014) and CLHLS (Yi, 2008). These cohort studies were designed from the angle of multidisciplines and were not intended for hearing and cognitive impairment analysis. This may account for the study design shortcomings in the included articles.

Even though speech perception is less affected due to high-frequency age-related hearing loss in the Sinitic language-speaking population, compared with the non-tonal language population, a significant association between ARHL and cognitive impairment was found in this meta-analysis. However, most of the enrolled studies adopted self-reported hearing loss, and with that administered audiometry, only a standard four-frequencies average was measured; the information on specific frequency ranges is not available. Furthermore, most of the included studies relied on cognitive screening instruments, which provide little domain-specific information on cognitive function. This may also explain why this meta-analysis did not observe the proposed difference in the relationship between hearing loss and cognitive decline among different language populations.

### Clinical Implications

Our findings confirm a significant association between untreated hearing loss, cognitive impairment, and dementia among Sinitic tonal language speakers. Given the higher prevalence of dementia and hearing loss in China, addressing these comorbid health conditions could have significant health and economic benefits. The first step should include hearing and cognitive screening assessments as part of clinical protocols for older adults aged 60 years and older in both hearing and memory clinics. Hearing health care professionals should explore the possibility of incorporating objective electrophysiological measurements into the hearing test battery, given their use in assessing hearing acuity in cognitively impaired older adults who are unable to provide accurate responses to behavioral pure tone audiometry. Little is known about clinical staff’s knowledge, attitudes, and practices in supporting hearing-impaired older adults with memory problems and the referral pathways between hearing clinics and memory clinics. Future studies need to determine these, and then to address any gaps that are identified.

## Conclusions

Our systematic review and meta-analysis of observational studies demonstrated a significant association between hearing loss and cognitive impairment or dementia in Sinitic language-speaking populations. This aligns with the previously published results of non-tonal language-speaking populations.

## Supplementary Material

igac078_suppl_Supplementary_Material

## Data Availability

All data generated or analyzed in this study are included in this published article and its supplementary information files; further inquiries can be directed to the corresponding author.
