# Supplementary material for: The Relationship of Age-Related Hearing Loss with Cognitive Decline and Dementia in a Sinitic Language-Speaking Adult Population: A Systematic Review and Meta-Analysis
Source: Innov Aging. 2022 Dec 20;7(1):igac078. doi: 10.1093/geroni/igac078 (PMC9976757; doi:10.1093/geroni/igac078)
Supplement: igac078_suppl_Supplementary_Material [file igac078_suppl_supplementary_material.docx]

**Online Supplementary Material**

eFig 1. Subgroup Forest plot by cognitive impairment or dementia, setting OR=as the effect size

eFig 2. Subgroup Forest plot by the type of hearing assessment, setting OR=as the effect size

eFig 3. Subgroup Forest plot by the type of sample size, setting OR=as the effect size

eFig 4. Subgroup Forest plot by the type of hearing assessment, setting regression coefficient as the effect size

eFig 5. Subgroup Forest plot by the type of sample size, setting regression coefficient as the effect size

eFig 6. Sensitivity analysis of included studies, setting regression coefficient as the effect size

eFig 7. Sensitivity analysis of included studies, setting OR=as the effect size

eTable 1. Quality assessment of the cohort studies

eTable 2. Quality assessment of the case-control studies

eTable 3. Quality assessment of the cross-sectional studies

eTable 4 PRISMA Checklist


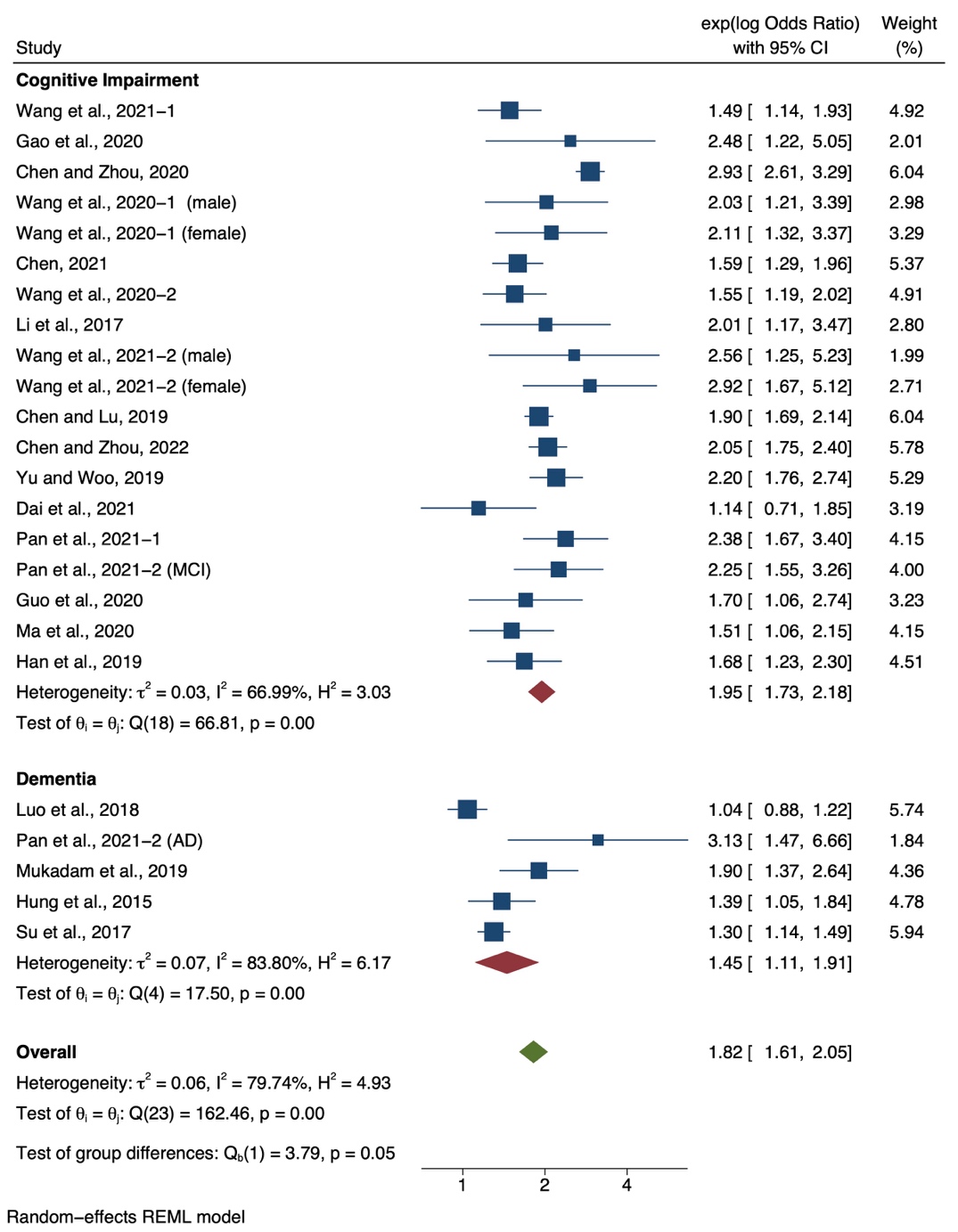


eFig 1. Subgroup Forest plot by cognitive impairment or dementia, setting OR=as the effect size


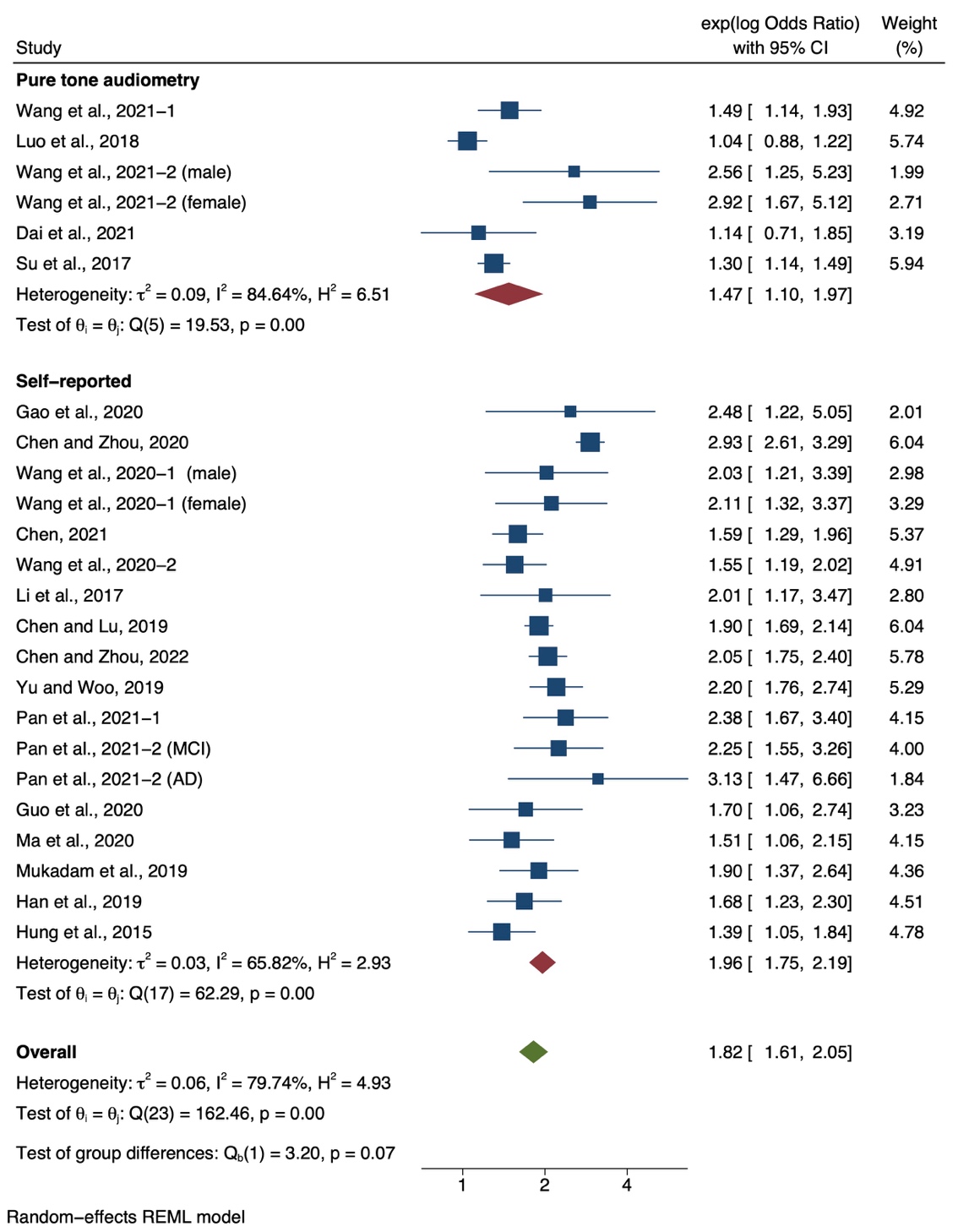


eFig 2. Subgroup Forest plot by the type of hearing assessment, setting OR=as the effect size


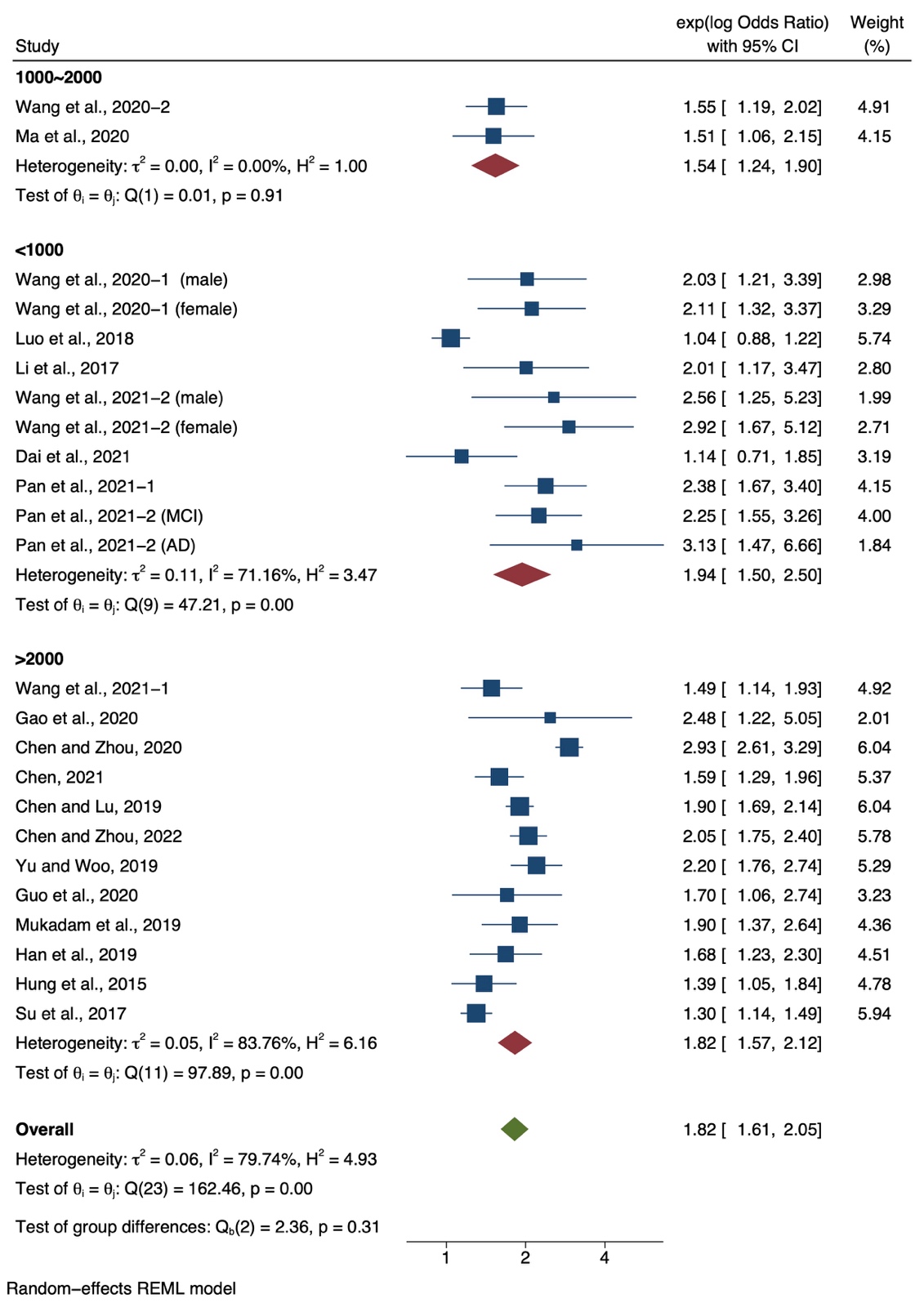


eFig 3. Subgroup Forest plot by the type of sample size, setting OR=as the effect size


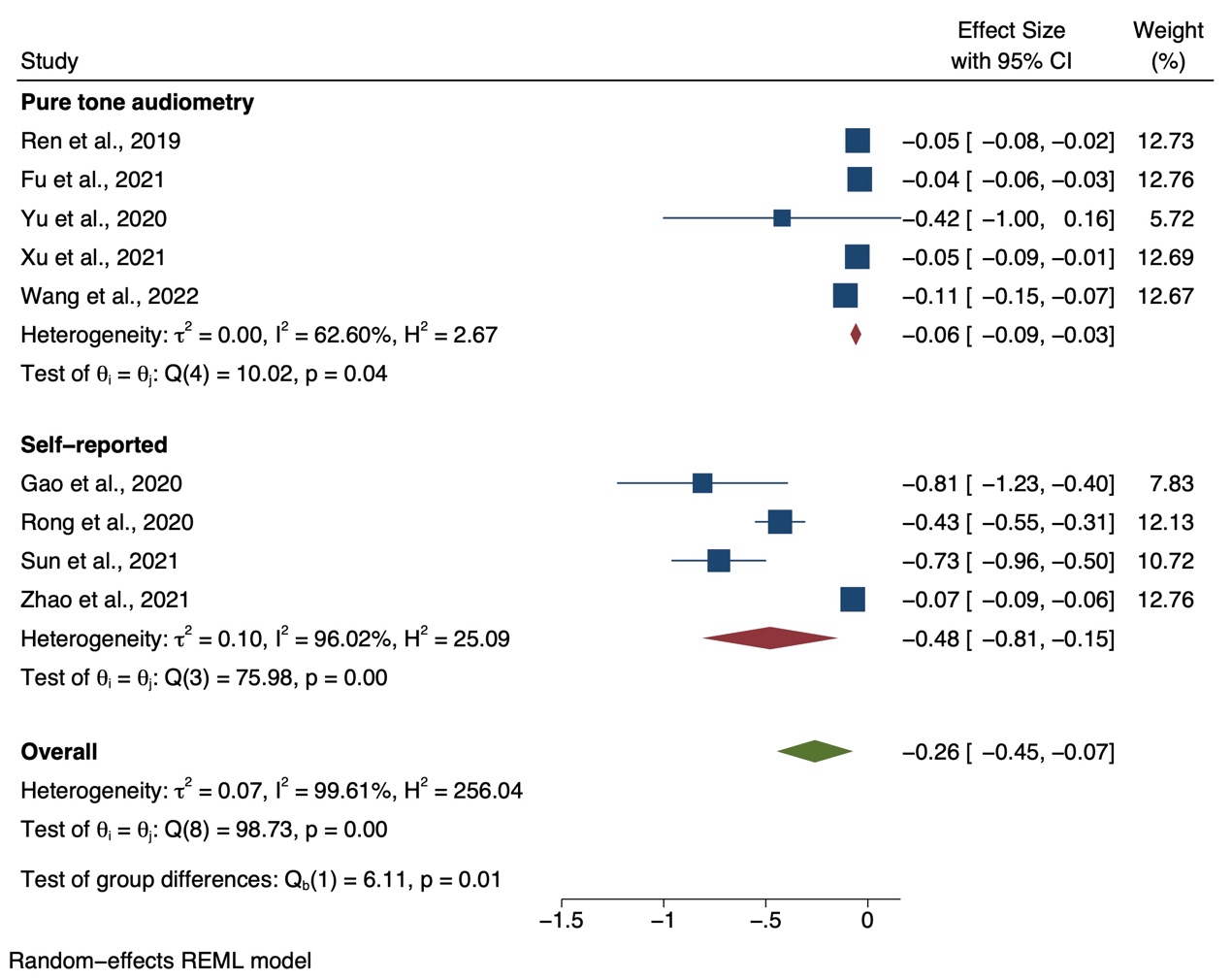


eFig 4. Subgroup Forest plot by the type of hearing assessment, setting regression coefficient as the effect size


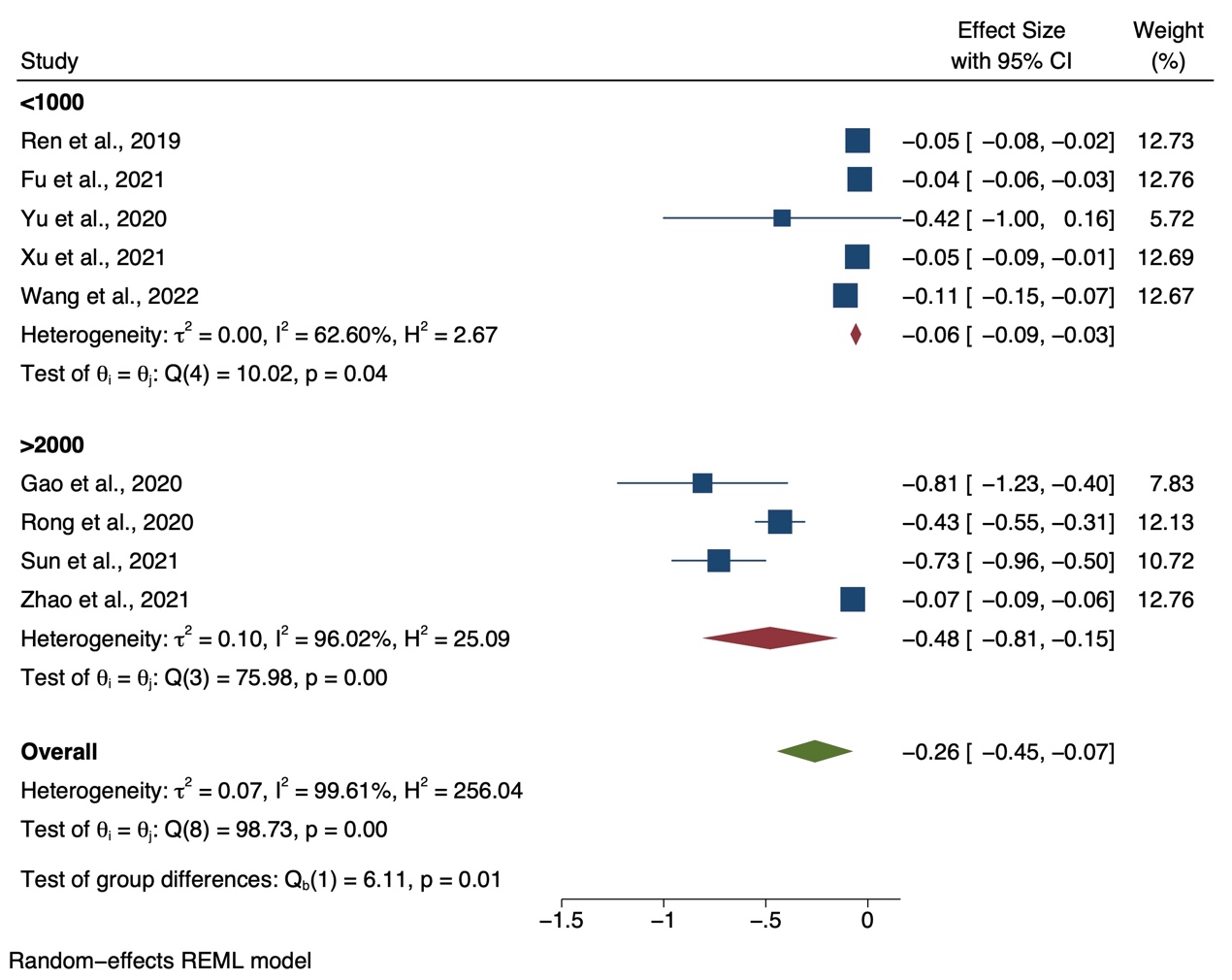


eFig 5. Subgroup Forest plot by the type of sample size, setting regression coefficient as the effect size


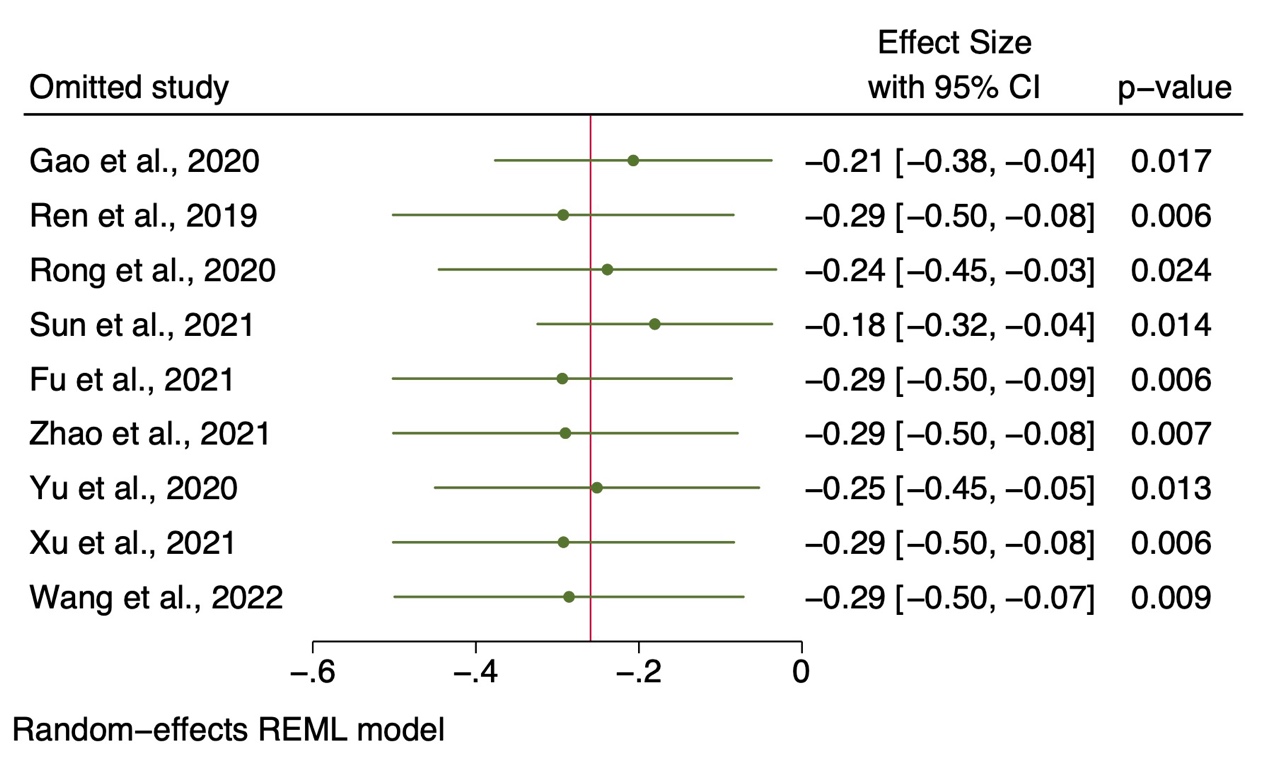


eFig 6. Sensitivity analysis of included studies, setting regression coefficient as the effect size


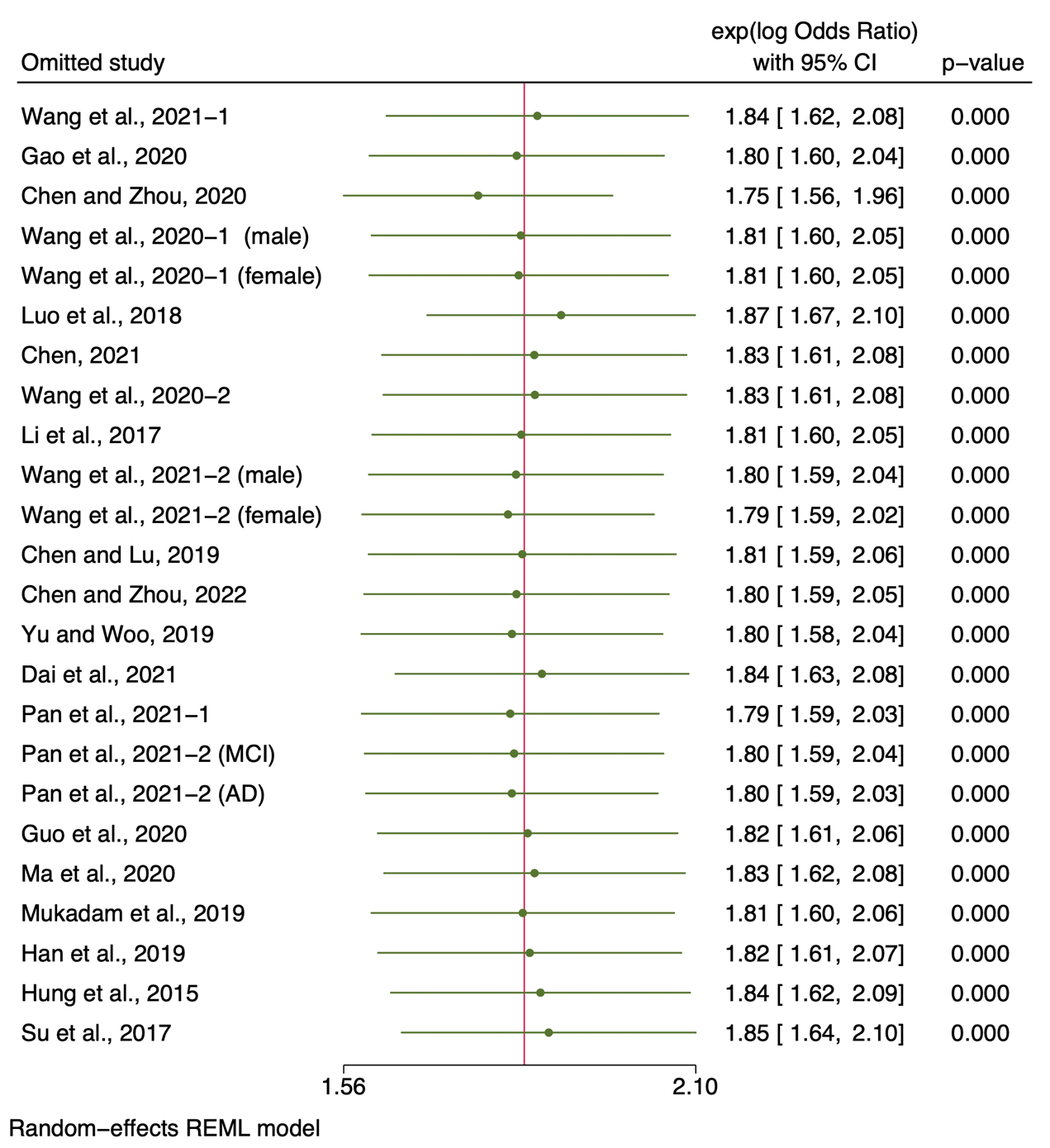


eFig 7. Sensitivity analysis of included studies, setting OR=as the effect size

eTable 1. Quality assessment of the cohort studies

| Studies | Selection | | | | | Outcome | | | Overall quality |
| --- | --- | --- | --- | --- | --- | --- | --- | --- | --- |
|  | Representativeness of the exposed cohort | Selection of the non-exposed cohort | Ascertainment of exposure | Demonstration that outcome of interest was not present at start of study | Comparability of cohorts on the basis of the design or analysis | Assessment | Follow-up long enough | Adequacy of follow up of cohorts |  |
| Gao et al., 2020 | 1 | 1 | 1 | 0 | 2 | 1 | 1 | 1 | Good |
| Chen and Zhou, 2020 | 1 | 1 | 1 | 0 | 2 | 1 | 1 | 1 | Good |
| Chen, 2021 | 1 | 1 | 1 | 1 | 2 | 1 | 1 | 1 | Good |
| Qiu et al., 2021 | 1 | 1 | 1 | 0 | 2 | 1 | 1 | 1 | Good |
| Chen and Lu, 2019 | 1 | 1 | 1 | 1 | 2 | 1 | 1 | 1 | Good |
| Yu and Woo, 2019 | 1 | 1 | 1 | 0 | 2 | 1 | 0 | 1 | Good |
| Ma et al., 2020 | 1 | 1 | 1 | 0 | 2 | 1 | 1 | 1 | Good |
| Su et al., 2017 | 1 | 1 | 1 | 0 | 2 | 1 | 1 | 1 | Good |

Protocol for quality assessment: according to their score in each section, articles were categorised as good, fair, or poor quality. Three or four stars in the selection domain, one or two stars in the comparability domain, and two or three stars in the outcome domain were considered good quality. Fair quality was defined as having two stars in the selection domain, one or two stars in the comparability domain, and two or three stars in the outcome domain. Poor quality was defined as having one or zero stars in the selection domain, zero stars in the comparability domain, and one or zero stars in the outcome domain.

eTable 2. Quality assessment of the case-control studies

| Studies No. | Selection | | | | | Outcome | | |  |
| --- | --- | --- | --- | --- | --- | --- | --- | --- | --- |
|  | Case definition adequate | Representativeness of the cases | Selection of Controls | Definition of Controls | Comparability of cases and controls on the basis of the design or analysis | Ascertainment of exposure | Same method of ascertainment for cases and controls | Non-Response rate | Overall quality |
| Dai et al., 2021 | 1 | 1 | 0 | 1 | 1 | 1 | 1 | 0 | Good |
| Yu et al., 2020 | 1 | 1 | 0 | 1 | 1 | 1 | 1 | 0 | Good |
| Hung et al., 2015 | 1 | 1 | 1 | 1 | 2 | 1 | 1 | 0 | Good |

Protocol for quality assessment: the same as in eTable 1.

eTable 3. Quality assessment of the cross-sectional studies

| Studies No. | Selection | | | | | Outcome | | |
| --- | --- | --- | --- | --- | --- | --- | --- | --- |
|  | Representativeness of the sample | Sample size | Non-respondents | Ascertainment of the exposure (risk factor) | Comparability Controlled for confounders | Assessment | Statistical test | Overall quality |
| Nicholas et al., 2021 | 1 | 0 | 0 | 2 | 1 | 2 | 1 | Good |
| Wang et al., 2021-1 | 1 | 1 | 0 | 2 | 1 | 2 | 1 | Good |
| Ren et al., 2020 | 0 | 0 | 0 | 2 | 1 | 1 | 1 | Fair |
| Wang et al., 2020-1 (male) | 1 | 0 | 1 | 1 | 1 | 2 | 1 | Good |
| Wang et al., 2020-1 (female) | 1 | 0 | 1 | 1 | 1 | 2 | 1 | Good |
| Luo et al., 2018 | 1 | 0 | 1 | 2 | 2 | 2 | 1 | Good |
| Ren et al., 2019 | 1 | 0 | 0 | 2 | 1 | 2 | 1 | Good |
| Ma et al., 2017 | 1 | 1 | 1 | 0 | 2 | 2 | 1 | Good |
| Wang et al., 2020-2 | 1 | 1 | 1 | 0 | 2 | 2 | 1 | Good |
| Li et al., 2017 | 0 | 0 | 0 | 2 | 1 | 2 | 1 | Fair |
| Wang et al., 2021-2 (male) | 1 | 0 | 0 | 2 | 2 | 2 | 1 | Good |
| Wang et al., 2021-2 (female) | 1 | 0 | 0 | 2 | 2 | 2 | 1 | Good |
| Rong et al., 2020 | 1 | 1 | 0 | 1 | 2 | 2 | 1 | Good |
| Sun et al., 2021 | 1 | 0 | 1 | 1 | 2 | 2 | 1 | Good |
| Fu et al., 2021 | 1 | 0 | 0 | 2 | 2 | 2 | 1 | Good |
| Zhao et al., 2021 | 1 | 0 | 1 | 1 | 2 | 2 | 1 | Good |
| Chen and Zhou, 2022 | 1 | 1 | 0 | 1 | 2 | 2 | 1 | Good |
| Pan et al., 2021-1 | 1 | 1 | 0 | 0 | 2 | 2 | 1 | Fair |
| Pan et al., 2021-2 (MCI) | 1 | 1 | 0 | 0 | 2 | 2 | 1 | Fair |
| Pan et al., 2021-2 (AD) | 1 | 1 | 0 | 0 | 2 | 2 | 1 | Fair |
| Guo et al., 2020 | 1 | 1 | 0 | 0 | 1 | 2 | 1 | Fair |
| Wang et al., 2019 | 0 | 0 | 0 | 2 | 1 | 2 | 1 | Fair |
| Xu et al., 2021 | 1 | 1 | 0 | 2 | 2 | 2 | 1 | Good |
| Diao et al., 2021 | 0 | 0 | 0 | 2 | 1 | 2 | 1 | Fair |
| Mukadam et al., 2019 | 1 | 1 | 0 | 1 | 1 | 2 | 1 | Good |
| Han et al., 2019 | 1 | 1 | 0 | 0 | 1 | 2 | 1 | Fair |
| Wang et al., 2022 | 0 | 0 | 0 | 2 | 2 | 2 | 1 | Fair |

Protocol for quality assessment: the same as in eTable 1.

eTable 4 PRISMA Checklist

| **Section and Topic** | **Item #** | **Checklist item** | **Location where item is reported** |
| --- | --- | --- | --- |
| **TITLE** | | |  |
| Title | 1 | Identify the report as a systematic review. | 1, title |
| **ABSTRACT** | | |  |
| Abstract | 2 | See the PRISMA 2020 for Abstracts checklist. | 2, abstract |
| **INTRODUCTION** | | |  |
| Rationale | 3 | Describe the rationale for the review in the context of existing knowledge. | 3, introduction |
| Objectives | 4 | Provide an explicit statement of the objective(s) or question(s) the review addresses. | 5, introduction |
| **METHODS** | | |  |
| Eligibility criteria | 5 | Specify the inclusion and exclusion criteria for the review and how studies were grouped for the syntheses. | 5-6, study eligibility |
| Information sources | 6 | Specify all databases, registers, websites, organisations, reference lists and other sources searched or consulted to identify studies. Specify the date when each source was last searched or consulted. | 6, information sources |
| Search strategy | 7 | Present the full search strategies for all databases, registers and websites, including any filters and limits used. | 6, search strategy |
| Selection process | 8 | Specify the methods used to decide whether a study met the inclusion criteria of the review, including how many reviewers screened each record and each report retrieved, whether they worked independently, and if applicable, details of automation tools used in the process. | 7, data management and study selection |
| Data collection process | 9 | Specify the methods used to collect data from reports, including how many reviewers collected data from each report, whether they worked independently, any processes for obtaining or confirming data from study investigators, and if applicable, details of automation tools used in the process. | 7, data management and study selection |
| Data items | 10a | List and define all outcomes for which data were sought. Specify whether all results that were compatible with each outcome domain in each study were sought (e.g. for all measures, time points, analyses), and if not, the methods used to decide which results to collect. | 7, data extraction |
|  | 10b | List and define all other variables for which data were sought (e.g. participant and intervention characteristics, funding sources). Describe any assumptions made about any missing or unclear information. | 7, data extraction |
| Study risk of bias assessment | 11 | Specify the methods used to assess risk of bias in the included studies, including details of the tool(s) used, how many reviewers assessed each study and whether they worked independently, and if applicable, details of automation tools used in the process. | 7, quality assessment and meta-bias(es) |
| Effect measures | 12 | Specify for each outcome the effect measure(s) (e.g. risk ratio, mean difference) used in the synthesis or presentation of results. | 8, meta-analytic approach |
| Synthesis methods | 13a | Describe the processes used to decide which studies were eligible for each synthesis (e.g. tabulating the study intervention characteristics and comparing against the planned groups for each synthesis (item #5)). | 8, synthesis of results |
|  | 13b | Describe any methods required to prepare the data for presentation or synthesis, such as handling of missing summary statistics, or data conversions. | 10, the effect size of pooled studies |
|  | 13c | Describe any methods used to tabulate or visually display results of individual studies and syntheses. | 8, synthesis of results |
|  | 13d | Describe any methods used to synthesize results and provide a rationale for the choice(s). If meta-analysis was performed, describe the model(s), method(s) to identify the presence and extent of statistical heterogeneity, and software package(s) used. | 8, meta-analytic approach |
|  | 13e | Describe any methods used to explore possible causes of heterogeneity among study results (e.g. subgroup analysis, meta-regression). | 8, heterogeneity and sensitivity analyses |
|  | 13f | Describe any sensitivity analyses conducted to assess robustness of the synthesized results. | 8, heterogeneity and sensitivity analyses |
| Reporting bias assessment | 14 | Describe any methods used to assess risk of bias due to missing results in a synthesis (arising from reporting biases). | 8, heterogeneity and sensitivity analyses |
| Certainty assessment | 15 | Describe any methods used to assess certainty (or confidence) in the body of evidence for an outcome. | 8, heterogeneity and sensitivity analyses |
| **RESULTS** | | |  |
| Study selection | 16a | Describe the results of the search and selection process, from the number of records identified in the search to the number of studies included in the review, ideally using a flow diagram. | 8-9, study Selection |
|  | 16b | Cite studies that might appear to meet the inclusion criteria, but which were excluded, and explain why they were excluded. | 8-9, study Selection |
| Study characteristics | 17 | Cite each included study and present its characteristics. | 9, study Characteristics |
| Risk of bias in studies | 18 | Present assessments of risk of bias for each included study. | 11, risk of bias within and across studies, eTable 1-3 |
| Results of individual studies | 19 | For all outcomes, present, for each study: (a) summary statistics for each group (where appropriate) and (b) an effect estimate and its precision (e.g. confidence/credible interval), ideally using structured tables or plots. | 11, sensitivity analyses. supplemental material efigure 6-7, |
| Results of syntheses | 20a | For each synthesis, briefly summarise the characteristics and risk of bias among contributing studies. | 11, risk of bias within and across studies |
|  | 20b | Present results of all statistical syntheses conducted. If meta-analysis was done, present for each the summary estimate and its precision (e.g. confidence/credible interval) and measures of statistical heterogeneity. If comparing groups, describe the direction of the effect. | 10-11, the effect size of pooled studies. Figure2-3. |
|  | 20c | Present results of all investigations of possible causes of heterogeneity among study results. | 11, risk of bias within and across studies |
|  | 20d | Present results of all sensitivity analyses conducted to assess the robustness of the synthesized results. | 11, sensitivity analyses. |
| Reporting biases | 21 | Present assessments of risk of bias due to missing results (arising from reporting biases) for each synthesis assessed. | 11, risk of bias within and across studies |
| Certainty of evidence | 22 | Present assessments of certainty (or confidence) in the body of evidence for each outcome assessed. | 11-12, subgroup analyses |
| **DISCUSSION** | | |  |
| Discussion | 23a | Provide a general interpretation of the results in the context of other evidence. | 12, discussion |
|  | 23b | Discuss any limitations of the evidence included in the review. | 13-14 |
|  | 23c | Discuss any limitations of the review processes used. | 15, paragraph 2 |
|  | 23d | Discuss implications of the results for practice, policy, and future research. | 16, conclusion |
| **OTHER INFORMATION** | | |  |
| Registration and protocol | 24a | Provide registration information for the review, including register name and registration number, or state that the review was not registered. | 5, methods |
|  | 24b | Indicate where the review protocol can be accessed, or state that a protocol was not prepared. | 6, search strategy |
|  | 24c | Describe and explain any amendments to information provided at registration or in the protocol. | NA |
| Support | 25 | Describe sources of financial or non-financial support for the review, and the role of the funders or sponsors in the review. | 16-17, funding |
| Competing interests | 26 | Declare any competing interests of review authors. | 16, competing interests |
| Availability of data, code and other materials | 27 | Report which of the following are publicly available and where they can be found: template data collection forms; data extracted from included studies; data used for all analyses; analytic code; any other materials used in the review. | 16, availability of data and materials |
